# Prognostic Factors and Clinical Characteristics of Duodenal Adenocarcinoma With Survival: A Retrospective Study

**DOI:** 10.3389/fonc.2021.795891

**Published:** 2021-12-15

**Authors:** Huapeng Sun, Yi Liu, Long Lv, Jingwen Li, Xiaofeng Liao, Wei Gong

**Affiliations:** ^1^ Department of General Surgery, Xiangyang Central Hospital, Hubei University of Arts and Science, Xiangyang, China; ^2^ Department of Medicinal Chemistry, School of Pharmacy, Hubei University of Chinese Medicine, Wuhan, China; ^3^ Department of Oncology, Xiangyang Central Hospital, Hubei University of Arts and Science, Xiangyang, China

**Keywords:** duodenal adenocarcinoma, histopathological phenotype, lymph node metastases, ATG12, overall survival

## Abstract

**Background:**

To evaluate the clinical risk factors that influence the overall survival in patients with duodenal adenocarcinoma (DA) after tumor resection.

**Methods:**

This study retrospectively analyzed 188 patients who underwent tumor resection for DA between January 2005 and June 2020 at Xiangyang Central Hospital.

**Results:**

The median survival of the patients who underwent resectional operation was 54 months, longer than of those who underwent palliative surgery (20.8 months) (2,916.17; 95% CI, 916.3−9,280.5; *p* < 0.001). Survival of non-ampullary duodenal carcinoma patients (50.3 months; 95% CI, 39.7−61.8) was similar to that of ampullary duodenal carcinoma patients (59.3 months; 95% CI, 38.6−66.7) but was significantly better than that of papillary adenocarcinoma patients (38.9 months; 95% CI, 29.8−54.8; *p* = 0.386). Those with intestinal-type ductal adenocarcinomas had a longer median overall survival than those with the gastric type (61.8 vs. 46.7 months; *p* < 0.01) or pancreatic type (32.2 months; *p* < 0.001). Clinical DA samples had significantly diverse expressions of ATG12, IRS2, and IGF2. Higher expressions of the ATG12 and IRS2 proteins were significantly correlated with worse survival. Multivariate Cox regression analysis revealed that lymph node metastasis (hazard ratio (HR), 6.44; 95% CI, 3.68−11.27; *p* < 0.0001), margin status (HR, 4.94; 95% CI, 2.85−8.54; *p* < 0.0001), and high expression of ATG12 (HR, 1.89; 95% CI, 1.17−3.06; *p* = 0.0099) were independent prognostic factors negatively associated with survival in patients undergoing curative resection. There was no survival difference between the groups with ampullary, non-ampullary, and papillary adenocarcinomas treated with adjuvant chemotherapy (*p* = 0.973).

**Conclusion:**

Gastric/pancreatic type, high expression of ATG12, lymph node metastases, and margin status were negative prognosticators of survival in patients with DAs than in those with tumor anatomical location. Curative resection is the best treatment option for appropriate patients.

## Introduction

Duodenal adenocarcinoma (DA) is a rare tumor, accounting for more than 50% of small bowel adenocarcinomas (SBAs) but only 0.5% of all gastrointestinal tumors. In many studies, the results of SBAs have been grouped together (1–3). DA is usually compared with colorectal cancer since the duodenum is located in the gastrointestinal tract and has similar molecules and possible phenotypic carcinogenesis (4, 5). However, left colon cancer (LCC) and right colon cancer (RCC) have distinguishable genomic patterns and clinical behaviors due to differences in the primary site of colon cancer, and patients with RCC or LCC generally exhibit different prognoses. Therefore, tumor location and genomic patterns are known prognostic factors in CRC and SBAs (6–8). It is clear that the results of each DA treatment modality need to be reported as a distinct entity.

Surgical resection remains the main treatment strategy for DAs. Through a more rigorous site-specific classification, many tumors previously believed to be “DAs” have been shown to contain different specific types, such as those derived from the ampulla of Vater, and papilla and non-ampullary cancers (3). To date, it is also recognized that ampullary carcinomas originating from the duodenal papilla have distinct characteristics. Although duodenal ampullary carcinoma is a typically large ulcer or adenocarcinoma of the intestinal type and usually has a better survival rate (5-year survival rate, 50%), it is often found in the papilla of duodenal carcinoma, and sclerosing circumferential tumor formation is a typical type of pancreatic biliary carcinoma. Although a small volume (average, 2.1 cm; 5-year survival rate, 6%), it still shows aggressive behavior. Furthermore, in addition to differences in incidence, the pathological assessment of tumor site involvement influenced outcomes (9, 10) and treatment (11, 12). Therefore, studying the biological characteristics of tumor sites, including their sensitivity to anticancer drugs, is crucial for improving the prognosis of patients with duodenal cancer.

In this study, we retrospectively analyzed and identified the clinicopathological and genomic features of DA based on single-center data and provided improved biological insight into the relationship between the different primary tumor sites of DAs and adjacent pancreatic cancers. In-depth genomic analysis has also revealed many targets, providing a theoretical basis to guide the application of targeted therapy in clinical care.

## Methods

### Study Design

This study was approved by the Institutional Review Board of the Xiangyang Central Hospital. The medical records of all patients who underwent duodenal carcinoma resection between January 2005 and June 2020 at Xiangyang Central Hospital were used. Non-invasive tumors (adenoma only cases) and tumors arising in the setting of familial adenomatous polyposis and Crohn’s disease were carefully excluded. With the use of these criteria, 62 cases qualified as non-ampullary DAs. For comparison, 57 ampullary adenocarcinomas and 69 papillary adenocarcinomas of the ductal were retrieved from the database during the same period of time and assessed for long-term follow-up. Archival H&E-stained glass slides of all pathologic specimens were reviewed separately by pathologists (Xiang Chunxiang) to determine the duodenal carcinoma phenotype. The classification of intestinal type, gastric type, or pancreatobiliary type was based on previously established criteria (13, 14). In instances where there was no uniform consensus regarding HP phenotype, H&E-stained slides were re-reviewed by the pathologists in a conference to reach a consensus.

Patients were excluded from the study if the cancer anatomical location of origin could not be determined or if they had indeterminate or mixed-type tumors. Furthermore, given the high proportion of intestinal and gastric subtypes observed in pancreatic cancers arising within intraductal papillary mucinous neoplasms of the pancreas (15), patients with these lesions were also excluded from the study.

Hospital and clinic medical records were reviewed for patient demographics, date of diagnosis, type and date of operation, use of neoadjuvant and adjuvant therapy, and follow-up; and overall survival (OS) was measured from the date of diagnosis to the date of death or last follow-up. Since this study was a retrospective review without direct patient contact, informed consent was not required.

### Patient Samples and Methods

Nine of the DA tissues were taken from the Xiangyang Central Hospital of patients for surgery from 2016 to 2019. The patients were aged 46−72 years, with an average age of 61 years. All nine patients underwent radical resection. There were two patients with stage II and seven patients with stage III. All pathological sections were reviewed by two senior pathologists to confirm the diagnosis. Three cases were well-differentiated, five cases had medium-to-low differentiation, seven cases had lymph node metastasis, and two cases had lymph node metastasis (**Supplementary Table S1**). All samples were stored at −80°C.

### Sample Preparation and Fractionation for Data-Dependent Acquisition Library Generation

iRT-Kits (Biognosys) were added to correct the relative retention time differences between runs with a volume proportion of 1:3 for iRT standard peptides versus sample peptides (16).

### Mass Spectrometry Data Analysis

MaxQuant (http://www.maxquant.org) is a widely used free software platform for protein identification and quantification developed by Max Planck Institutes for high-resolution MS data. The data-dependent acquisition (DDA) library was executed using this software for identification and served as a spectrum library for subsequent data-independent acquisition (DIA) analysis. The analysis used the raw data as input files, set the corresponding parameters and databases, and then performed identification and quantitative analysis. Identified peptides that satisfied false discovery rate (FDR) ≤1% were used to construct the final spectral library.

The DIA data were analyzed using iRT peptides for retention time calibration. Then, based on the target−decoy model applicable to SWATH-MS, false-positive control was performed with FDR 1%; therefore, significant quantitative results were obtained.

Proteins were defined as differentially expressed if the fold change between intestinal type, gastric type, and pancreatobiliary type group based on the fold change >1.5 and P-value < 0.05, as the criterion for the significant difference provided within the MS stats package. Gene ontology (GO) analysis and Kyoto Encyclopedia of Genes and Genomes (KEGG) pathway analysis were adopted to clarify the significant biological functions of aberrantly expressed proteins. Protein–protein interaction (PPI) networks were created for these proteins using the STRING database (http://string-db.org/).

### Immunohistochemical Analysis

Immunohistochemistry was performed using a polymer-based detection system (Envision+; Dako, Carpinteria, CA, USA) with mouse monoclonal antibodies according to the manufacturer’s instructions, as previously described [20]. Immunohistochemical (IHC) staining evaluation were determined based on the consensus of at least two reviewing study pathologists at multi-headed microscopes without knowledge of clinical information. The percentage of cells showing cytoplasmic (MUC2, MUC5AC, MUC6, CK7, and CK20), apical membranous or cytoplasmic (MUC1), and nuclear (CDX2) labeling was evaluated. Only cases with >25% or ≤25% immunoreactive cells were regarded as high or low for invasive protein status (IRS2, IGF2, and ATG12), the lack of nuclear staining in DA was interpreted as an abnormal result. The antibodies were purchased from Santa Cruz Biotechnology (Santa Cruz, CA, USA).

### Statistical Analysis

A software program (SPSS, version 25.0; IBM) was used for statistical analysis and data management. The means of continuous variables with normal distributions were compared using a two-tailed t*-*test. Non-parametric continuous variables were compared using the Mann−Whitney test or Kruskal−Wallis test. Survival analysis was performed using the Kaplan−Meier method, and comparisons were made using the log-rank test. Multivariable Cox proportional hazards regression analyses were performed using statistically significant univariate parameters with *p* < 0.05 as the initial entry criterion. Continuous variables were split at the medians for these analyses. The significance of the protein abundance changes was calculated using the non-parametric Student’s t-test with Bonferroni multiple test correction. A two-tailed test with *p* < 0.05 was considered significant. Graphs were prepared using GraphPad Prism (version 7.0; GraphPad Software, San Diego, CA, USA). Statistical significance was defined as *p* ≤ 0.05.

## Result

### Patient Characteristics

A total of 201 patients with DA who underwent surgical treatment were retrospectively studied (**Table 1**). The median age was 60 years (range, 33–75 years). Twenty-four patients underwent pylorus-sparing pancreaticoduodenectomy (12.8%), 30 patients underwent segmental duodenectomy (16%), and 108 patients underwent classical pancreaticoduodenectomy (57.4%). Possible curative surgery was performed in 162 patients (86.2%), and palliative care (gastrojejunostomy or double bypass) was performed in 26 patients (13.8%). Preoperative biliary drainage was required in 23 patients due to severe jaundice. Thirteen patients were lost to follow-up (six in the active treatment group and seven in the palliative treatment group). Therefore, 188 of 201 patients could be included in the long-term survival analysis. After surgical treatment, 101 of the 188 patients (53.7%) received adjuvant chemoradiotherapy.

**Table 1:** General characteristics of non-ampullary duodenal carcinomas compared with both ampullary carcinomas and duodenal papillary adenocarcinomas.

Characteristics	Non-ampullary duodenal carcinoma	Ampullary carcinoma	Duodenal papillary adenocarcinomas	p^†^	p^††^
	(n = 57)	(n = 62)	(n = 69)		
Mean age (years)	61.3 (59.2–63.4)	60 (57.9–62.3)	60.2 (58.1–62.4)	0.42	0.91
Gender				0.58	0.58
F	36 (63.2%)	36 (58.1%)	47 (68.1%)		
M	21 (36.8%)	26 (41.9%)	22 (31.9%)		
Tumor size (cm)				1	0.86
<3 cm	32 (56.1%)	35 (56.5%)	40 (58%)		
>3 cm	25 (43.9%)	27 (43.5%)	29 (42%)		
Histologic grade				0.85	0.07
Well or moderately differentiated	24 (42.1%)	28 (45.2%)	41 (59.4%)		
Low or poorly differentiated	33 (57.9%)	34 (54.8)	28 (40.6%)		
LN status				0.39	0.07
Positive	11 (19.3%)	17 (27.4%)	24 (34.8%)		
Negative	46 (80.7%)	45 (72.6%)	45 (65.2%)		
Margin status				1	0.8
Negative	50 (87.8%)	54 (87.1%)	59 (85.6%)		
Positive	7 (12.2%)	8 (12.9%)	10 (14.4%)		
Perineural invasion				<0.01	1
Present	6 (10.5%)	28 (45.2%)	7 (10.1%)		
Not present	51 (89.5%)	34 (54.8%)	62 (89.9%)		
TNM stage				0.71	0.072
I to II stage	31 (54.4%)	36 (58.1%)	35 (50.7%)		
III to IV stage	26 (45.6%)	26 (41.9%)	34 (49.3%)		
Surgery procedure				1	0.62
Radical resection	50 (87.7%)	54 (87.1%)	58 (84.1%)		
Palliative care	7 (12.3%)	8 (12.9%)	11 (15.9%)		
Adjuvant treatment				0.86	0.28
Yes	28 (49.1%)	32 (51.6%)	41 (59.4%)		
No	29 (50.9%)	30 (48.4%)	28 (40.6%)		
CEA				0.41	0.35
Normal	28 (49.1%)	34 (54.8%)	39 (56.5%)		
High	11 (19.3%)	15 (24.2%)	16 (23.2%)		
Inconclusive	18 (31.6%)	13 (21%)	14 (20.3%)		
CA-199				0.41	0.47
Normal	27 (47.4%)	33 (53.2%)	37 (53.6%)		
High	13 (22.8%)	17 (27.4%)	18 (26.1%)		
Inconclusive	17 (29.8%)	12 (19.4%)	14 (20.3%)		

F, female; M, male.

^†^ *p* comparison between non-ampullary duodenal carcinoma and ampullary carcinoma.

^††^ *p* comparison between non-ampullary duodenal carcinoma and duodenal papillary adenocarcinomas.

**Table 1** summarizes the anatomical distribution of DA. The most common tumor site was the duodenal papilla (n = 69), followed by the ampullary (n = 57) and non-ampullary (n = 62). In terms of mean tumor size, non-ampullary duodenal carcinoma was similar to ampullary duodenal carcinoma and papillary adenocarcinoma (range: <3 vs. >3 cm, 56.1% vs. 43.9% for non-ampullary; 56.5% vs. 43.5% for ampullary, *p* = 1; and 58% vs. 42% for the papilla, *p* = 0.86). According to the 7th edition of the American Joint Committee on Cancer (AJCC)-TNM classification, 45.7% of the cases were mainly stage III and stage IV (45.6% for non-ampullary vs. 41.9% for ampullary, *p* = 0.71; and 49.2% for the papilla, *p* = 0.72). Most of the patients who were treated with palliative care had stage III and IV disease (stage I, 0%; stage II, 0%; stage III, 12%; and stage IV, 88%). The positive rate for lymph nodes was 27.7% (19.3% in the non-ampullary group and 27.4% in the ampullary group, *p* = 0.39; 34.8% in the papillary group, *p* = 0.07). The marginal positive rate of non-ampullary carcinoma (12.2%) was similar to that of ampullary carcinoma (12.9%) but lower than that of papillary adenocarcinoma (14.4%; *p* = 0.8). The frequency of perineural invasion was 21.8% (10.5% for non-ampullary, 45.2% for ampullary, *p* < 0.01, and 10.1% for the papilla, *p* = 1). In total, 101 patients with serum tumor markers were not elevated before surgery. Forty-eight patients (25.5%) and 42 patients (22.3%) had abnormal increases in carbohydrate antigen 199 (CA-199) and carcinoembryonic antigen (CEA), respectively (**Table 1**).

**Figure 1:**
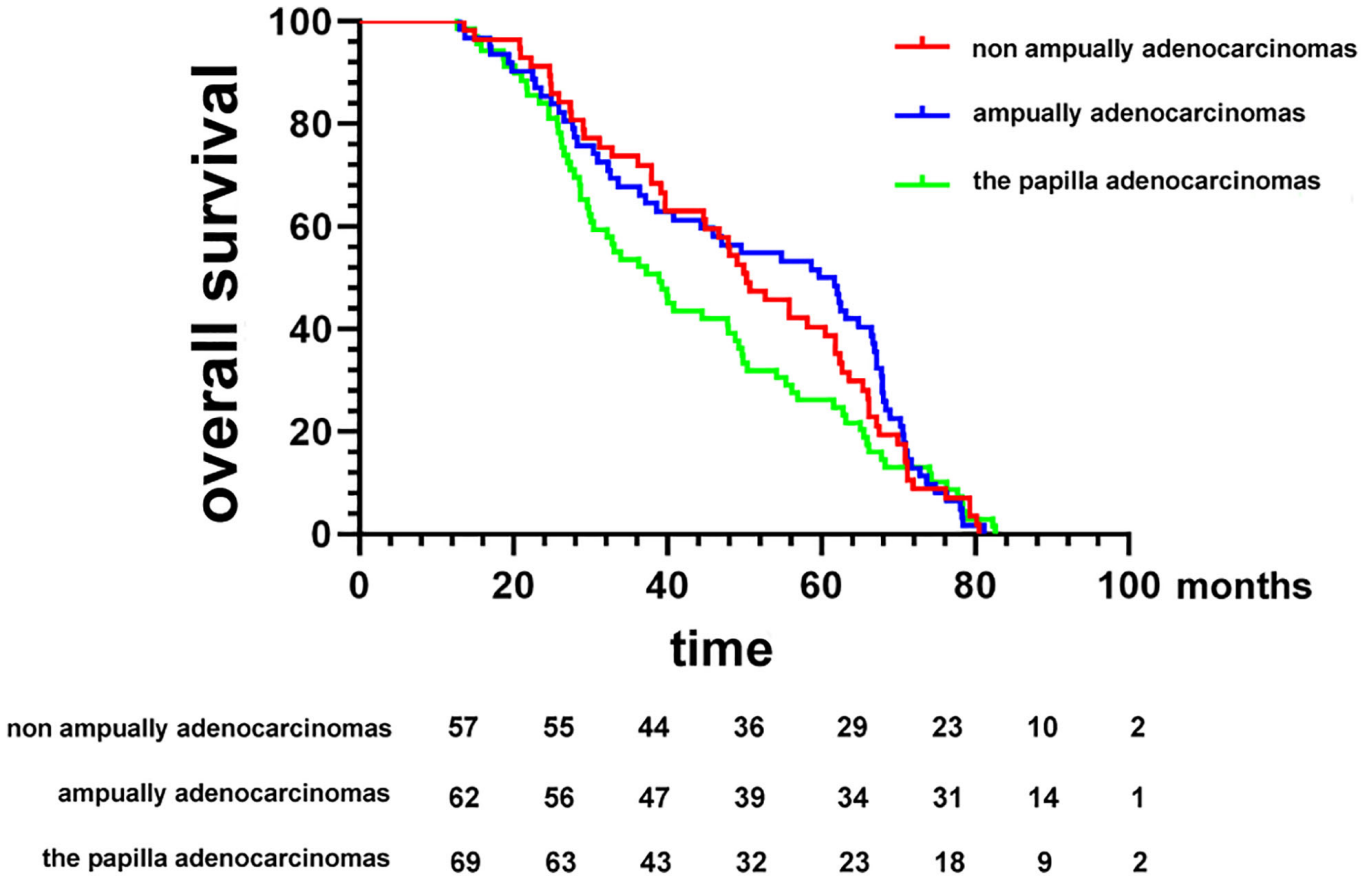
Kaplan−Meier survival curves comparing the overall survival of patients after resection of duodenal adenocarcinomas grouped by tumor anatomical location of origin.

In this study, the Kaplan−Meier analysis showed that patients with pancreatic adenocarcinoma had the shortest survival time as compared with patients with DA at diverse anatomical sites (median OS, 27.9 months; *p* < 0.01) (data not shown). However, survival time also varied greatly between patients with cancers originating in different anatomical locations, with a median OS of 38.9 months (95% CI, 29.8−48.8 months) for the papilla group, 59.3 months (95% CI, 38.6−66.7 months) for the ampulla group, and 50.3 months (95% CI, 39.7−61.8 months) for the non-ampullary group (*p* = 0.386; **Figure 1**).

### Immunoprofile in the Comparison of Non-Ampullary Duodenal Carcinomas With Both Ampullary Carcinomas and Papillary Carcinomas

**Figure 2:**
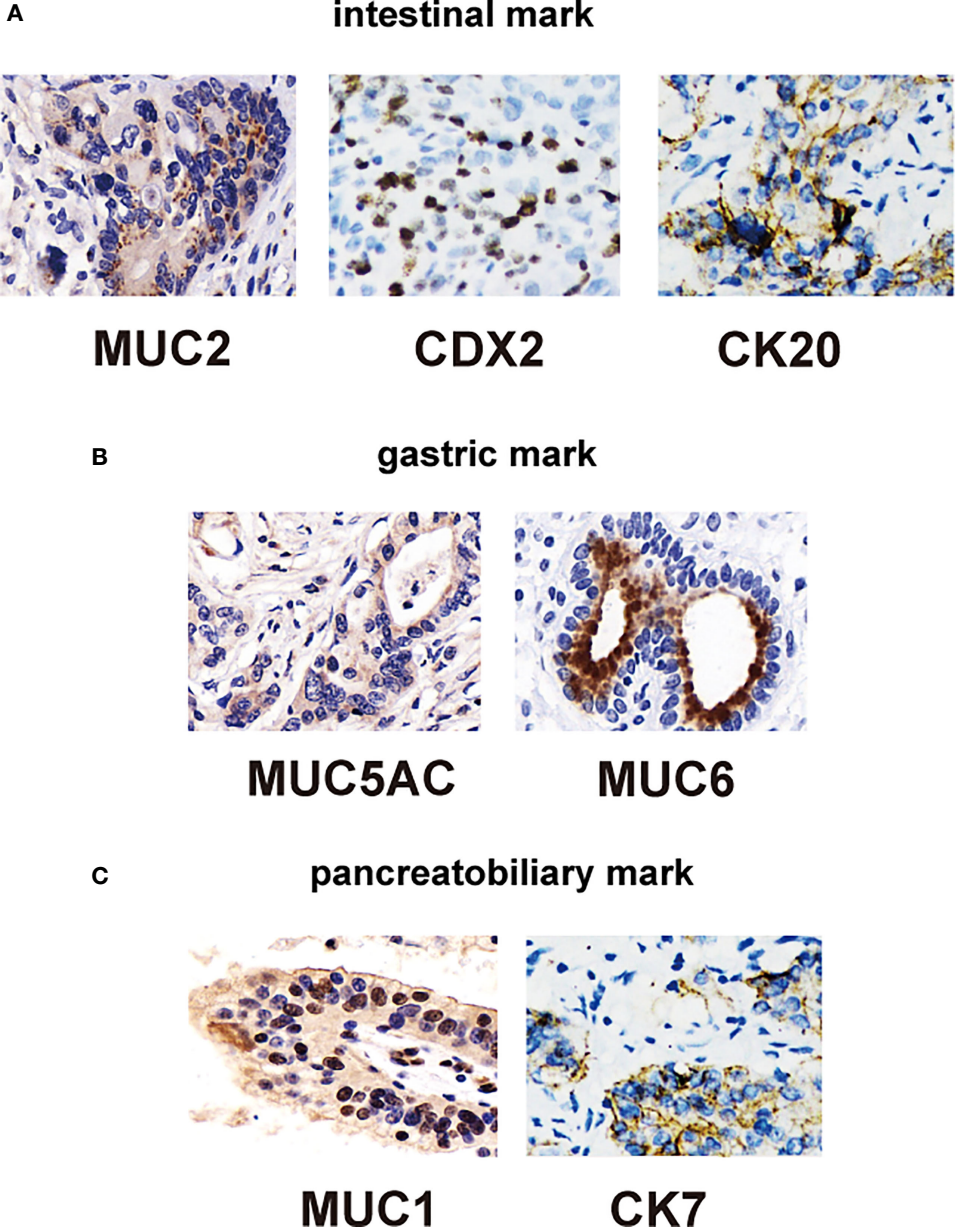
Immunohistochemical staining of duodenal carcinomas tissue. **(A)** Immunohistochemical stains of intestinal type: positive for CDX2 (left), CK20 (middle), and MUC2 (right) markers. **(B)** Immunohistochemical stains of gastric type: positive for MUC5AC (middle) and MUC6 (right) markers. **(C)** Immunohistochemical stains of pancreatic type: positive for MUC1 (middle) and CK7 (right) markers.

Ang et al. (17) recently proposed IHC criteria for the classification of DA into intestinal, pancreatic, biliary, and gastric types. **Figures 2A–C** compares the clinicopathological factors associated with DA. IHC markers that are typically expressed consistently in intestinal types are relatively low in frequency in non-ampullary duodenal carcinomas (MUC2, CK20, and CDX2), whereas those of gastric and pancreatobiliary types appear to be fairly common (CK7, MUC1, MUC5AC, and MUC6) (**Figure 2**). In addition, 40.3% of the 62 cases in the ampullary group were non-intestinal type (22.6% pancreatic type and 17.7% gastric type). Of the 69 cases of papillary adenocarcinoma, 58% were classified as non-intestinal type (fit pancreaticobiliary, 42%; and gastric, 16%) (**Table 2**); in general, the histological characteristics of intestinal-type adenocarcinoma are not as aggressive as those of gastric and pancreatic-type adenocarcinomas. Intestinal-type adenocarcinoma had significantly less lymph node involvement, less perineural vascular involvement, and a lower histological grade. In contrast, gastric- and pancreatic-type adenocarcinomas are larger and more likely to show advanced stage III or IV disease (**Table 3**).

**Table 2:** Expression of the immunohistochemical markers of non-ampullary duodenal carcinomas compared with both ampullary carcinomas and duodenal papillary adenocarcinomas.

Tumor anatomical Location	Pancreatobiliary markers		Gastric markers		Intestinal markers		
	MUC1	CK7	MUC5AC	MUC6	MUC2	CK20	CDX2
Non-ampullary duodenal carcinoma	11		15		31		
Ampullary carcinoma	14		11		37		
Duodenal papillary adenocarcinomas	29		11		29		

**Table 3:** Clinicopathological factors by histopathological phenotype.

Characteristics	Intestinal type	Gastric type	Pancreatobiliary type	*p*†	*p*††
	(n = 97)	(n = 37)	(n = 54)		
Mean age (years)	59.7 (58–61.3)	61.1 (57.8–64.4)	61.6 (59.5–63.8)	0.38	0.18
Gender				0.42	1
F	60 (61.9%)	26 (70.3%)	33 (61.1%)		
M	37 (38.1%)	11 (29.7%)	21 (38.9%)		
Tumor size (cm)				0.70	0.74
<3 cm	55 (56.7%)	23 (62.2%)	29 (53.7%)		
>3 cm	42 (43.3%)	14 (37.8%)	25 (46.3%)		
Histologic grade				0.70	0.61
Well or moderately differentiated	46 (42.1%)	19(45.2%)	28 (59.4%)		
Low or poorly differentiated	51 (57.9%)	18 (54.8)	26 (40.6%)		
LN status				0.11	0.004
Positive	18 (19.3%)	12 (27.4%)	22 (34.8%)		
Negative	79 (80.7%)	25 (72.6%)	32 (65.2%)		
Margin status				0.15	1
Negative	82 (87.8%)	35 (94.6%)	46 (85.6%)		
Positive	15 (12.2%)	2 (5.4%)	8 (14.4%)		
Perineural invasion				0.34	0.53
Present	18 (10.5%)	10 (45.2%)	13 (10.1%)		
Not present	79 (89.5%)	27 (54.8%)	41 (89.9%)		
TNM stage				0.56	0.089
I to II stage	58 (59.8%)	20 (54.1%)	24 (44.4%)		
III to IV stage	39 (40.2%)	17 (45.9%)	30 (55.6%)		
Surgery procedure				0.02	0.007
Radical resection	91 (93.8%)	29 (78.3%)	42 (77.8%)		
Palliative care	6 (6.2%)	8 (21.7%)	12 (22.2%)		
Adjuvant treatmen				0.698	0.498
Yes	53 (54.6%)	22 (59.5%)	26 (48.1%)		
No	44 (45.4%)	15 (40.5%)	28 (51.9%)		

F, female; M, male.

†*p* comparison between non-ampullary duodenal carcinoma and ampullary carcinoma.

††*p* comparison between non-ampullary duodenal carcinoma and duodenal papillary adenocarcinomas.

Pancreatic-type adenocarcinoma was more likely to have similar margins (eight patients [14.4%]) than intestinal-type cancers (15 patients [12.2%]; *p* = 1) and gastric-type cancers (eight patients [8.4%]; *p* = 0.15). Among the 188 patients with known details of adjuvant therapy, chemotherapy was the main treatment (101 patients [53.7%]), including 53 patients with intestinal type, 22 patients with gastric cancer (*p* = 0.698), and 26 patients with pancreatic type (*p* = 0.498). These results did not show significant differences in the proportion of patients undergoing chemotherapy among all groups (54.6% intestinal type, 59.5% gastric type, and 48.1% pancreatic type).

**Figure 3:**
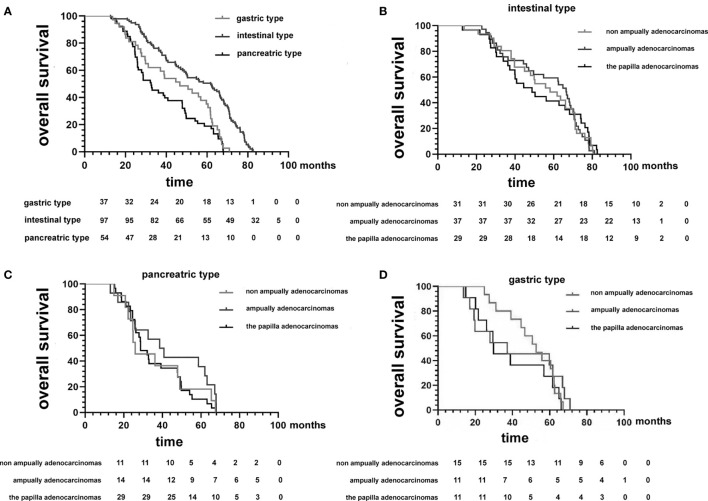
**(A)** Kaplan−Meier survival curves comparing the overall survival of patients after resection of duodenal adenocarcinomas grouped by histopathological phenotype. **(B–D)** Kaplan−Meier survival curves comparing the overall survival of patients after resection of duodenal adenocarcinomas grouped by histopathological phenotype between the three tumor anatomical locations of origin.

In the Kaplan−Meier analysis, we found that patients were grouped by histological differentiation rather than anatomical location and that survival in patients with pancreatic type was almost identical to that in patients with gastric-type adenocarcinoma (median OS, 32.2 vs. 46.7 months; *p* = 0.66); the survival time of patients in both groups was significantly shorter than that of patients with intestinal-type adenocarcinoma (median OS, 61.8 months; *p* < 0.001) (**Figure 3A**). Furthermore, in patients with gastric-type adenocarcinoma, we were able to determine the median OS, which was similar to that at different anatomical sites (37.2 months for ampullary, 52.7 months for non-ampullary, and 29.8 months for papillary; *p* = 0.44) (**Figure 3B**). There was also no significant difference in OS between intestinal-type adenocarcinoma at different anatomical locations (ampullary, 66.5 months; non-ampullary, 58.2 months; and papillary, 48.8 months; *p* = 0.716) (**Figure 3C**). The OS was at least twice as long in patients with intestinal-type adenocarcinoma as compared with those with pancreatic-type adenocarcinoma at different anatomical locations (ampullary, 39.7 months; non-ampullary, 25.9 months; and papillary, 28.7 months; *p* = 0.649) (**Figure 3D**). Considering the similarity of survival in patients with pancreatic and gastric tumors, we combined the two clinicopathological phenotypes for further multivariate analysis.

### Genetic and Molecular Biology Considerations

**Figure 4:**
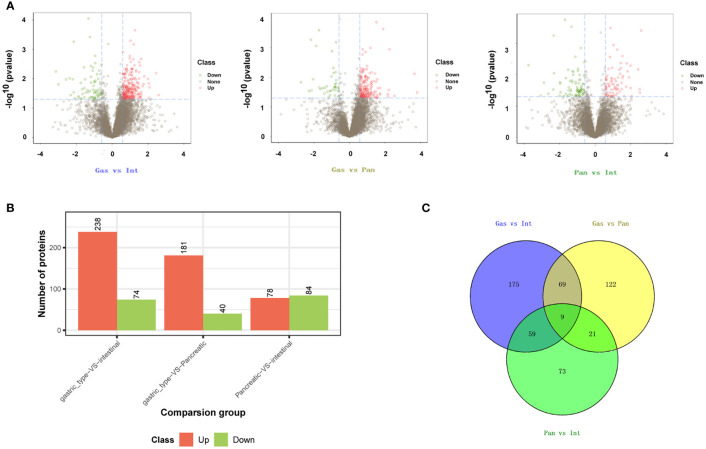
Identification of differentially expressed genes (DEGs) in duodenal adenocarcinomas. DEGs in duodenal adenocarcinoma for the group of gastric type vs. intestinal type, gastric type vs. pancreatic type, and pancreatic type vs. intestinal type. **(A)** Volcano plot. **(B)** Bar histogram. **(C)** Venn diagram.

Due to the rarity of DA, little has been published on oncogenesis and clinicopathological features (18). To elucidate the biology behind proteomic changes in pathological types, we identified a cohort of tissues from three patients with DA with gastric, intestinal, and pancreatic types (**Supplementary Table S1**). To ensure validity and accuracy, we performed follow-up biological and statistical analyses. The correlation coefficient of the quality control (QC) sample indicates the stability of the entire experimental operation and the reliability of the test results (**Supplementary Table S2** and **Figure S1**). In this study, we selected proteins at constant levels found in >50% of the samples for subsequent statistical and bioinformatic analyses, including 9,300 proteins and 85,309 peptides. Among the three groups for the gastric, pancreatic, and intestinal types, after the results` of expressed proteins were standardized, we identified 312 differentially expressed proteins (DEPs) in the gastric vs. pancreatic group, 221 DEPs in the gastric vs. intestinal group, and 162 DEPs in the pancreatic vs. intestinal group (**Figures 4A, B**). In these three datasets, there were 30 proteins, 68 proteins, and 78 proteins that overlapped in the gastric vs. pancreatic group and the pancreatic vs. intestinal group, in the gastric vs. intestinal group and the pancreatic vs. intestinal group, and in the gastric vs. pancreatic group and the gastric vs. intestinal group, respectively. Nine proteins overlapped in the three groups (**Figure 4C**).

**Figure 5:**
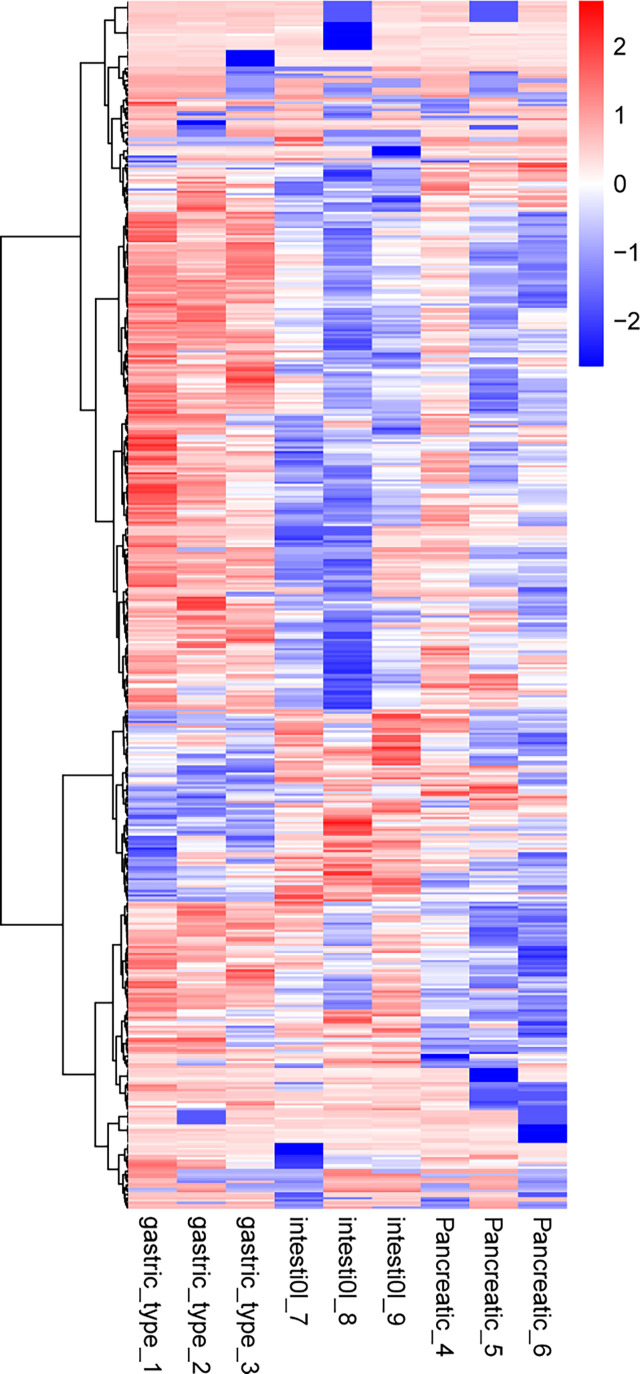
Cluster analysis of differentially expressed proteins in the group of gastric type, pancreatic type, and intestinal type. The hierarchical clustering results are represented as a tree heat map, with the ordinate representing significantly differentially expressed proteins and the abscissa representing sample information. Significant differences in protein expression in the different numerical expression quantities (log_2_ expression) of the samples with different colors are shown on the heat map, where red or blue represents significantly upregulated or downregulated proteins, and gray represents no quantitative information for proteins. ATG12 represents the protein name of Q94817, IGF2 represents the protein name of p03814, and IRS2 represents the protein name of Q9Y4H2.

Hierarchical cluster analysis was performed on 497 and 198 dysregulated proteins, and the results of the heat maps provided protein profiles for the gastric, pancreatic, and intestinal groups (**Figure 5**). Thirty-six DEPs were analyzed in the gastric vs. intestinal, gastric vs. pancreatic, and pancreatic vs. intestinal groups in combination with known protein functions, and the biological behaviors of 21 proteins were found to be correlated with tumor. Further screening for proteins related to tumor growth, invasion, and metastasis, and regarding *IRS2*, *IGF2*, and its role-related gene *ATG12*, it was found that *IRS2* and *IGF2* were highly expressed in the gastric group than in the intestinal and pancreatic groups, and *ATG12* was highly expressed in the gastric and pancreatic groups compared with the intestinal group (**Supplementary Table S3**).

**Figure 6:**
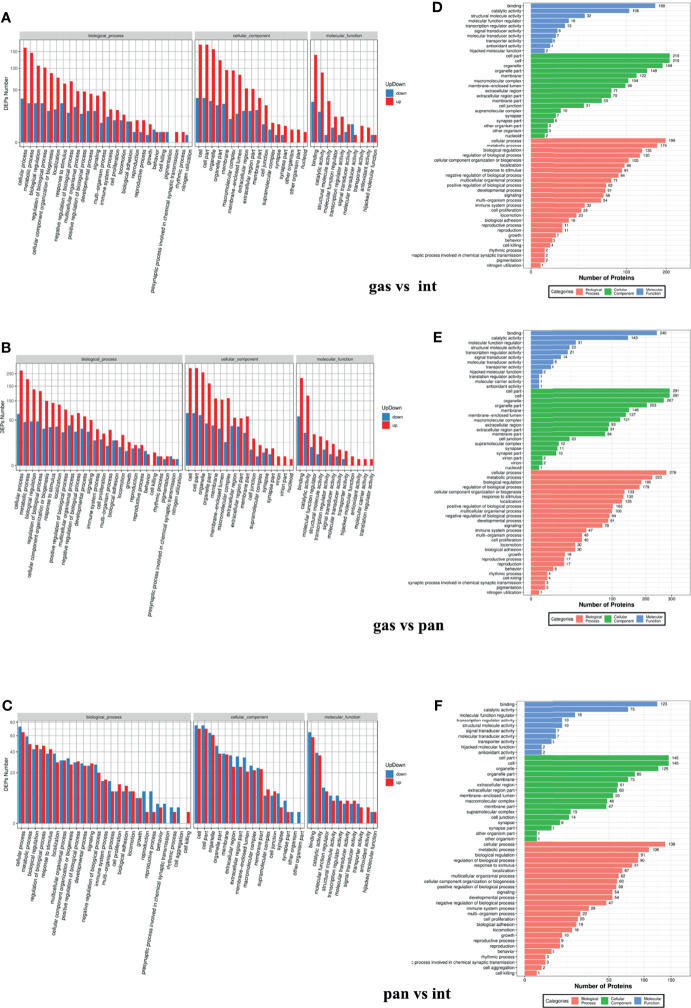
Gene ontology functional enrichment analysis of gastric vs. intestinal, gastric vs. pancreatic, and pancreatic vs. intestinal differentially expressed proteins. **(A–C)** Represent biological processes analysis, cellular components analysis, and molecular function analysis. **(D–F)** The number of differentially expressed proteins enriched in each entry.

**Figure 7:**
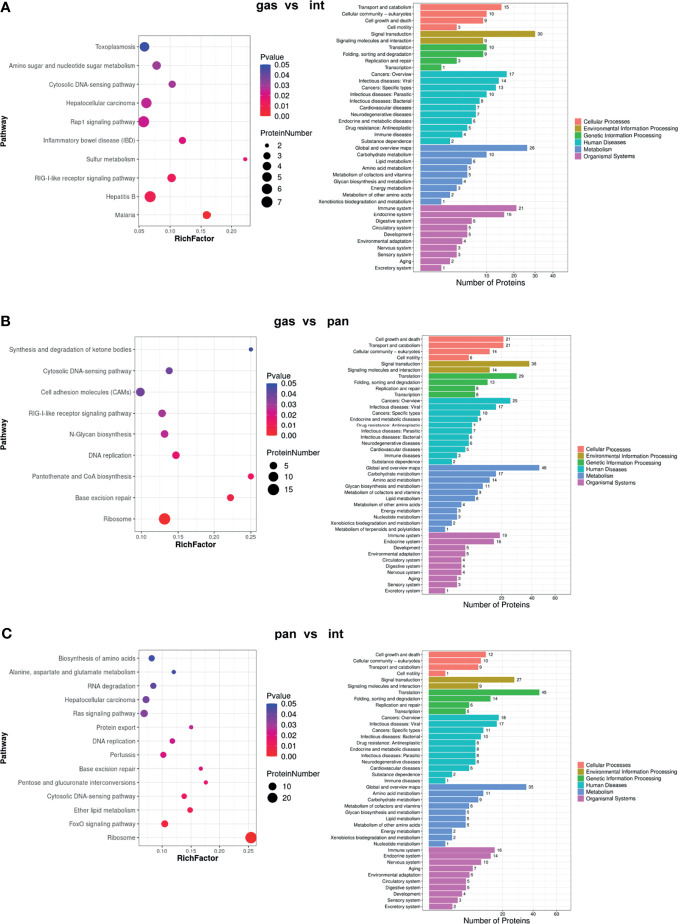
Kyoto Encyclopedia of Genes and Genomes (KEGG) functional enrichment analysis of gastric vs. intestinal **(A)**, gastric vs. pancreatic **(B)**, and pancreatic vs. intestinal **(C)**, differentially expressed protein signaling pathways. IRS2, IGF2, and ATG12 proteins are present mainly in cancer metabolic pathways (containing MAPK, FoxO, and RAS signaling pathways).

**Figure 8:**
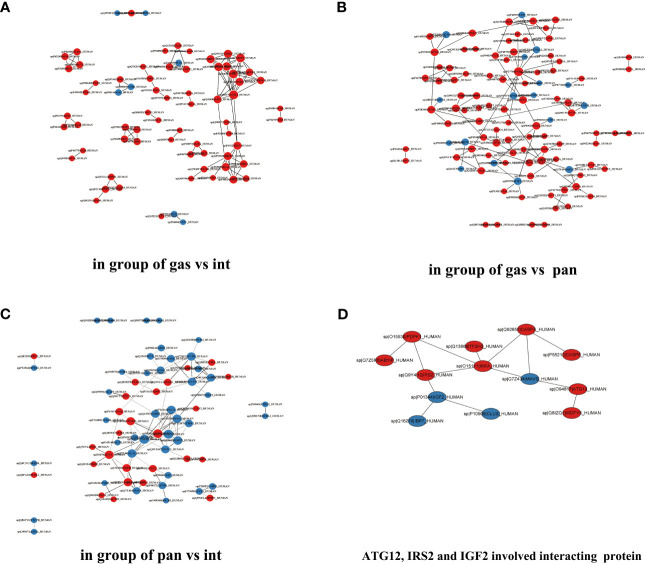
Differentially expressed protein interaction networks in the group of gastric type vs. intestinal type **(A)**, gastric type vs. pancreatic type **(B)**, and pancreatic type vs. intestinal type **(C)**. **(D)** ATG12, IGF2, and IRS2 involved interacting proteins.

DAVID was used to carry out functional and pathway enrichment analyses to analyze the biological classification of DEPs. GO functional annotation was performed for all proteins screened in the project. Differences in biological process (BP), molecular function (MF), and cellular component (CC) protein expression were the greatest in the gastric and intestinal groups, pancreatic and intestinal groups, and gastric and pancreatic groups. The results focused on cellular processes, cellular parts, and binding (**Figures 6A**–**F**). IRS2, IGF2, and ATG12 are mainly enriched in the metabolic processes of BP and MF protein binding. The whole DEPs in the gastric vs. intestinal group, pancreatic vs. intestinal group, and gastric vs. pancreatic group were mainly concentrated in signal transduction, translation, global and overview maps, and cancer; the overview is shown by the KEGG pathway analysis (**Figures 7A**–**C**). The IRS2, IGF2, and ATG12 proteins were present mainly in cancer metabolic pathways, including the MAPK, FOXO, and RAS signaling pathways. The PPI network was also presented to show the correlations among common DEPs (**Figures 8A**–**D**).

**Figure 9:**
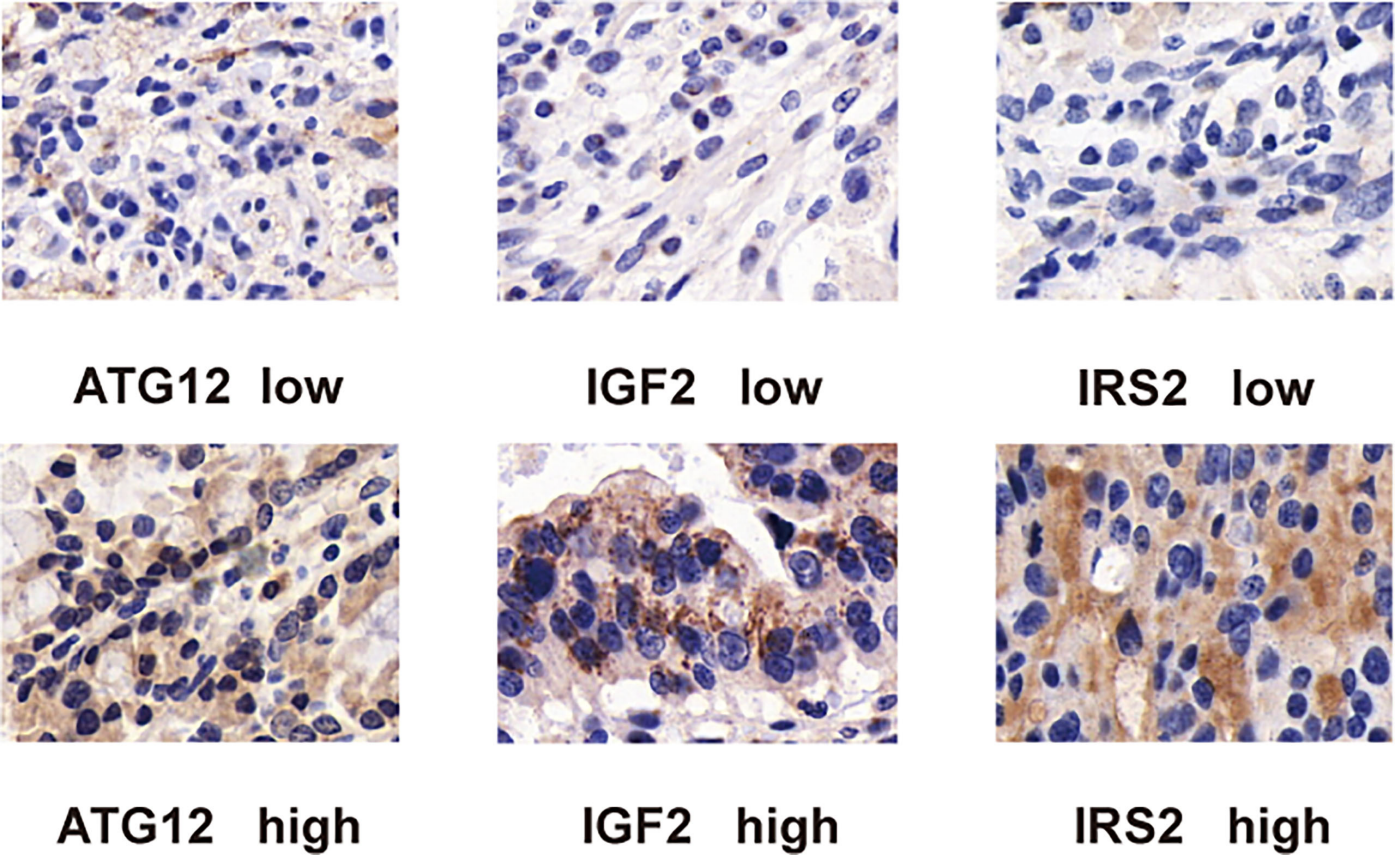
Immunohistochemical staining of duodenal carcinoma tissue stained with **(A)** ATG12, **(B)** IRS2, and **(C)** IGF2 antibodies. For all antibodies, proteins are detected in both the cytoplasm and the nuclei of tumor cells.

**Figure 10:**
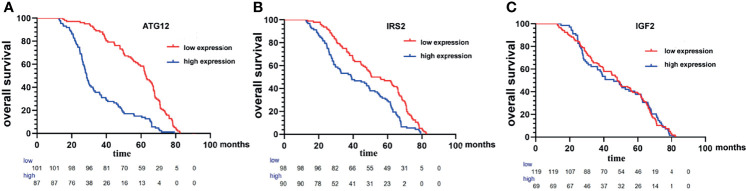
Kaplan−Meier survival curves of the overall survival of patients with duodenal adenocarcinoma with high expression of ATG12 **(A)**, IRS2 **(B)**, and IGF2 **(C)** compared with those patients with low expression of ATG12, IRS2, and IGF2.

To confirm the above findings, we assessed the expression of IRS2, IGF2, and ATG12 by IHC in tumor tissues from 188 patients (**Figure 9**). In our study, OS analysis of hub proteins was performed using the Kaplan–Meier curves. DA patients with high expression of IRS2 and ATG12 showed worse OS (*p* < 0.05, **Figures 10A, B**). The OS was numerically but not statistically significant in high or low expression levels of IGF2 (*p* > 0.05, **Figure 10C**). Together, these results suggest that the inactivation pathway of IRS2 or ATG12 is associated with OS in patients with DA.

### Multivariable Cox Regression Analysis of Survival

In the univariate analysis, surgery palliative care, histopathological phenotype (gastric and pancreatic type), presence of lymph node metastases, higher TNM stage, tumor metastasis, and high expression of ATG12 and IRS2 were all associated with poor prognosis (**Table 4**). However, age, sex, tumor size, tumor location, differentiation, perineural invasion, and adjuvant chemoradiotherapy were not associated with survival. However, in multivariate analysis, only histologic grade, radical surgical resection, nodal status, HP phenotype, and high expression of ATG12 but not IRS2 (**Table 4**) were significant prognostic indicators. In multivariate analysis, tumor anatomical location was no longer associated with survival.

**Table 4:** Results of the Cox regression to identify independent potential variables influencing overall survival of patients undergoing resection for duodenal adenocarcinoma.

Characteristics	Univariable analysis		Multivariable analysis	
	Hazard ratio	P-value	Hazard ratio	P-value
Age	1.33 (0.99–1.79)	0.058		
<60 years				
>60 years				
Gender	1.04 (0.77–1.40)	0.808		
Male				
Female				
Tumor size	0.94 (0.71–1.26)	0.697		
<3 cm				
>3 cm				
Tumor location	1.14 (0.85–1.55)	0.383		
Ampullary carcinoma				
Non-ampullary and papillary adenocarcinomas				
Differentiation	0.86 (0.64–1.15)	0.304	0.94 (0.70–1.27)	0.693
Well or moderately differentiated				
Low or poorly differentiated				
Histopathological phenotype	2.96 (2.11–4.15)	<0.01	2.97 (1.85–4.77)	<0.0001
Intestinal type				
Gastric and pancreatobiliary type				
Perineural invasion	1.09 (0.76–1.55)	0.636	1.20 (0.83–1.74)	0.338
Present				
Not present				
Lymph node metastasis	54.13 (29.03–100.90)	<0.001	6.44 (3.68–11.27)	<0.0001
Positive				
Negative				
TNM stage	3.72 (2.63–5.28)	<0.001	0.87 (0.52–1.46)	0.602
I and II				
III and IV				
Margin status	6.72 (3.40–13.27)	<0.01	4.94 (2.85–8.54)	<0.0001
Negative				
Positive				
Surgery procedure	2,916.17 (916.3–9,280.5)	<0.0001	4.78 (2.59–8.81)	<0.0001
Radical resection				
Palliative resection				
Adjuvant therapy	1.01 (0.75–1.34)	0.973		
No				
Yes				
IRS2 expression	1.82 (1.34–2.47)	0.0001	.93 (0.61–1.41)	0.72
Low				
High				
ATG12 expression	4.27 (2.99–6.10)	<0.0001	1.89 (1.17–3.06)	0.0099
Low				
High				

Analyzed as a continuous variable.

## Discussion

DA is a rare cancer with an incidence of fewer than 0.5/100,000 people (19). Due to the low incidence and prevalence of DA, few studies have been published, and relevant survival factors remain controversial (20). In the present study, we retrospectively analyzed tumor location, clinicopathological features, proteomic characterization, treatment, and outcomes of metastasis in patients with surgically resected DA at our institution. Univariate analysis and subsequent multivariate Cox regression analysis showed that patients with DA resection who underwent palliative surgical excision, had worse tumor grade, and had TNM stage had shorter OS; and clinicopathological characteristics and regional LN metastasis were also associated with prognosis (21). However, larger and multicenter studies are needed to investigate the mechanistic associations between prognostic factors such as lymph node status, surgical resection type, adjuvant therapy, and survival.

Interestingly, our results show that patients with ampullary, non-ampullary, and papillary adenocarcinomas differed in survival and response to adjuvant therapy at each tumor anatomical site after tumor resection. Compared with ampullary adenocarcinoma, non-ampullary adenocarcinoma and papillary adenocarcinoma are not very similar, and the survival rates of ampullary adenocarcinoma, non-ampullary adenocarcinoma, and papillary adenocarcinoma may be different, but the difference is not statistically significant. In contrast, there were significant differences in the anatomical location of intestinal-, gastric-, and pancreatic-type adenocarcinomas for patient survival, suggesting that they may represent different diseases (21, 22). The prognosis of the pancreatic/gastric type is worse than that of the intestinal type, and the clinicopathological subtype may be an independent prognostic factor (23). Importantly, the proportion of patients with AJCC stage III/IV tumors with gastric/pancreatic-type tumors was significantly higher. Therefore, the current study emphasizes that in DA classified according to the AJCC staging system, the anatomical extent of the disease strongly influences the prognosis (24). More importantly, duodenal carcinoma of the pure or almost exclusively gastric/pancreatic-type adenocarcinoma is similar to pancreatic ductal adenocarcinoma in that it is aggressive and has worse OS because pancreatic adenocarcinoma is more likely to present as a migration status. As mentioned earlier, determining the exact anatomical site is often arduous and imprecise. Pathological analysis, usually performed under the naked eye or microscope, determines the central location of the tumor, suggesting that grouping cancers in the field by clinicopathological phenotype rather than anatomical location can better estimate survival and may guide clinical decision-making. In addition to behavioral differences, cancers in different regions of this relatively small region may have different reasons and molecular mechanisms in the future, each of which may require different treatments.

Radical resection and the Whipple procedure (pancreaticoduodenectomy) provided the best opportunity for the successful treatment of DA patients. Palliative surgery is reserved for cases in which the disease is diagnosed in an advanced stage, and radical surgery does not confer any survival benefit. Lymph node metastasis and distant metastasis in surgery were strongly associated with negative outcomes. Therefore, adequate lymph node dissection is important for survival prediction and treatment. At the same time, our results may also influence the surgical treatment of these patients.

Several additional limitations need to be emphasized, particularly those inherent in retrospective database analysis. As with all observational studies, coding errors and data omissions are possible. The role of postoperative adjuvant chemotherapy and/or radio/chemotherapy in DA is unclear (25, 26). Early studies have shown that neoadjuvant chemoradiotherapy can improve local control before surgery, but without benefit for OS (27). The Phase II prospective trial at the M.D. Anderson Cancer Center supported the results, with response rates approaching 50% when oxaliplatin and capecitabine were used in combination (28). In our retrospective study, adjuvant chemotherapy was not associated with improved OS. However, some authors suggest that gem-based therapies are not often effective for carcinomas of intestinal origin, but it may be reasonable to apply current guidelines for the treatment of pancreatic cancer to pancreatic-type adenocarcinoma (21). Therefore, there is a tendency to change the treatment of patients with DA based on whether the tumor shows a histological phenotype in the pancreatic, gastric, or intestinal types (29), and the results suggested when considering treating patients with different types of tumors with chemotherapy, it may be best to consider different pathological-based regimens because these drugs are generally not effective for cancer of different origins (30). The adjuvant or neoadjuvant effect of systemic chemotherapy warrants further study.

Using laser capture microdissection combined with an optimized high-sensitivity proteomic pipeline, we quantified up to 5,000 unique protein groups per sample from as little as 5,000 cancer cells. This approach enabled compartment-resolved proteomic analysis of gastric, intestinal, and pancreatic types and revealed biological signatures. Our results highlight the molecular heterogeneity of DA and reveal that the tumor proteome is relatively stable in individual patients, as recently observed in breast cancer (27). There were a total of 673 DEPs, including 172 upregulated and 501 downregulated proteins in DA samples, of which 36 were differentially co-expressed in the three groups. After analyzing the biological behavior of the 36 proteins, we found that the ATG12 and IRS2 proteins were highly expressed in the pancreatic and gastric types, and the IGF2 proteins were highly expressed in the intestinal and gastric types. In the OS analysis, it was concluded that the prognosis for patients with DA was poor if ATG12 and IRS2 were highly expressed but not IGF2. However, the high expression level of ATG12 but not IRS2 was correlated with OS in terms of key patient symptoms such as clinicopathological characteristics, the presence of lymph node metastasis, and degree of tumor differentiation, or whether the patient had distant metastasis. Therefore, ATG12 is most likely to play a role in the malignant biological behavior of DA, which is consistent with our proteomics conclusions, which fully aroused our interest in ATG12. Interestingly, Mafficini et al. recently established a subtype of patients with DA that probably benefited from therapies targeting the ERBB, PI3K, or WNT signaling pathways (31). Perhaps, as our understanding of the molecular and genetic origins of DA improves, more targeted treatment strategies will contribute to better outcomes.

As a unique proteome entity, DA, unlike CRC and GC, should prompt further research into the optimal clinical treatment of this rare carcinoma. The identification of multiple clinically relevant proteomic alterations and mutation profiles in populations with limited treatment options and poor prognoses is encouraging.

## Data Availability Statement

Proteomics raw date are available in iproX (www.iprox.org) under the accession number IPX0003803000.

## Ethics Statement

This retrospective study was approved by the ethics committee of the Xiangyang Central Hospital (Reference-no 2020-018) and conducted in accordance with the Declaration of Helsinki. Written informed consent referred to routine diagnostics and academic assessment of the archived biopsy specimen as well as transfer of clinical data was obtained from all patients.

## Author Contributions

WG conceived the study; HS, YL, and LL designed and performed the majority of experiments, assisted by JL; XL contributed reagents and analytic tools. WG and XL analysed the data and wrote the manuscript. All authors contributed to the article and approved the submitted version.

## Funding

This work was supported by the National Science Foundation of China (Nos. 81772491 and 81373433) and the Youth Top Talent Project of Hubei Medical.

## Conflict of Interest

The authors declare that the research was conducted in the absence of any commercial or financial relationships that could be construed as a potential conflict of interest.

## Publisher’s Note

All claims expressed in this article are solely those of the authors and do not necessarily represent those of their affiliated organizations, or those of the publisher, the editors and the reviewers. Any product that may be evaluated in this article, or claim that may be made by its manufacturer, is not guaranteed or endorsed by the publisher.

## Supplementary Material

Supplementary Figure 1 Quantitative values of protein **(A)** and peptide **(B)** in the samples.
