# Supplementary figures and images for: Prognostic Factors and Clinical Characteristics of Duodenal Adenocarcinoma With Survival: A Retrospective Study
Source: Front Oncol. 2021 Dec 15;11:795891. doi: 10.3389/fonc.2021.795891 (PMC8715708; doi:10.3389/fonc.2021.795891)

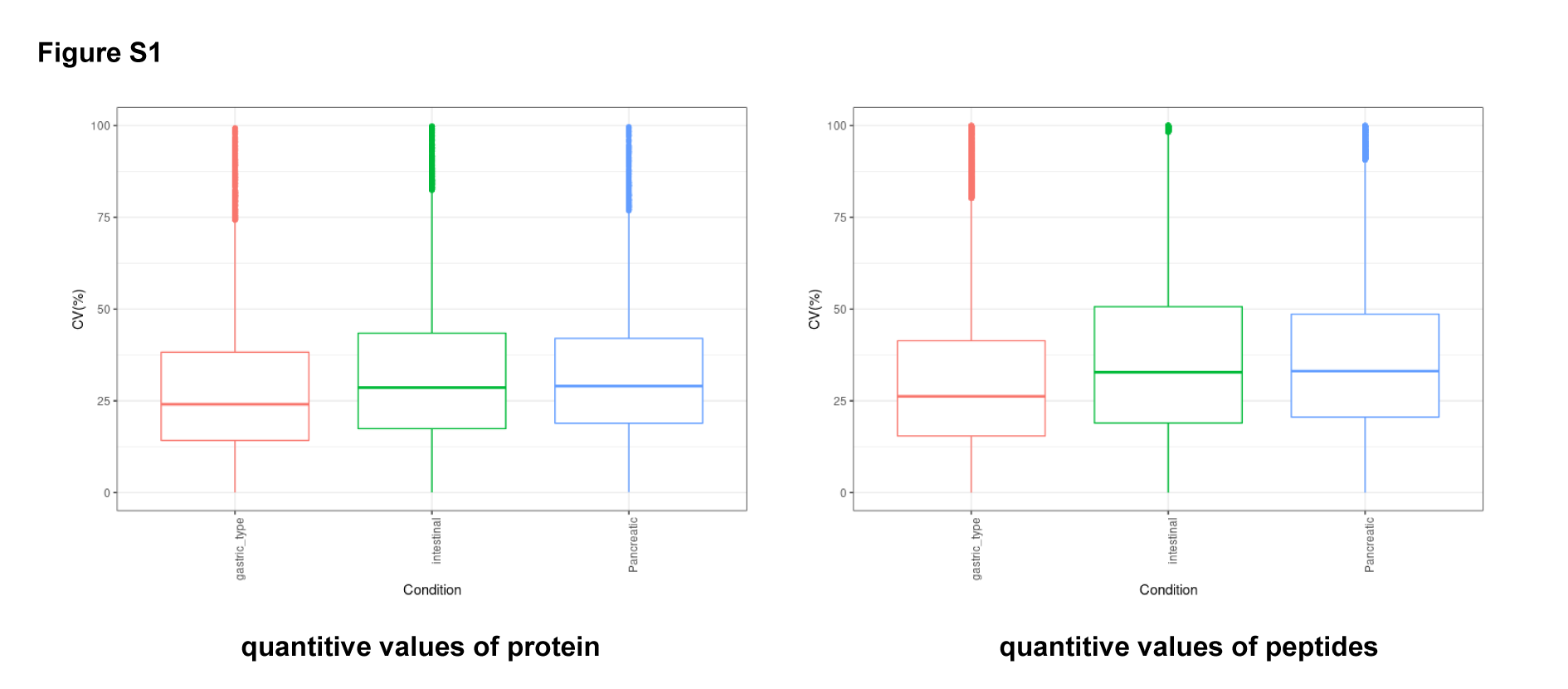

Supplement: Supplementary Figure 1 — Quantitative values of protein (A) and peptide (B) in the samples. [file Image_1.tif]
