# Supplementary material for: Prognostic Factors and Clinical Characteristics of Duodenal Adenocarcinoma With Survival: A Retrospective Study
Source: Front Oncol. 2021 Dec 15;11:795891. doi: 10.3389/fonc.2021.795891 (PMC8715708; doi:10.3389/fonc.2021.795891)
Supplement: Supplementary file 2 [file Table_1.docx]

Table SⅠ. Perform proteomic analysis on tumor samples from gastric type, intestinal type, and pancreatic type in a cohort of 3 DA patients

| **Patient** | **Histopathologic phenotype** | **AJCC stage .7th** | **LN** | **Dx Age** | **Adjuvant chemotherapy** | **Surgery procedure** | **sex** | **OS** | **tumor location** |
| --- | --- | --- | --- | --- | --- | --- | --- | --- | --- |
| 1 | gastric type | III | N1 | 68 | yes | radical resection | male | 22.5m | ampullary |
| 2 | gastric type | II | N0 | 58 | no | radical resection | male | 50.7m | Non ampullary |
| 3 | gastric type | III | N1 | 46 | yes | radical resection | female | 25.8m | the papilla |
| 4 | pancreatic type | III | N1 | 54 | yes | radical resection | male | 29.6m | the papilla |
| 5 | pancreatic type | III | N1 | 72 | yes | radical resection | female | 32.8m | ampullary |
| 6 | pancreatic type | III | N1 | 60 | no | radical resection | female | 40.8m | Non ampullary |
| 7 | intestinal type | II | N0 | 56 | no | radical resection | male | 49.6m | ampullary |
| 8 | intestinal type | III | N1 | 70 | yes | radical resection | female | 24.9m | Non ampullary |
| 9 | intestinal type | III | N1 | 66 | yes | radical resection | male | 44.4m | Non ampullary |

LN: Lymph node involvement status.N1 positive lymph nodes, N0 negative lymph node

Dx Age: diagnosis age
