# Supplementary material for: Prognostic Factors and Clinical Characteristics of Duodenal Adenocarcinoma With Survival: A Retrospective Study
Source: Front Oncol. 2021 Dec 15;11:795891. doi: 10.3389/fonc.2021.795891 (PMC8715708; doi:10.3389/fonc.2021.795891)
Supplement: Supplementary file 3 [file Table_2.docx]

Table SⅡ. overview of quantitative results for each sample

Name peptide number protein number

Gastric type1 41118 6420

Gastric type2 38291 6248

Gastric type3 36302 6047

Intestinal type 7 36148 5995

Intestinal type 8 31306 5616

Intestinal type 9 38519 6279

Pancreatic type 4 40332 6396

Pancreatic type 5 32389 5801

Pancreatic type 6 32326 5709
