# Supplementary material for: Prognostic Factors and Clinical Characteristics of Duodenal Adenocarcinoma With Survival: A Retrospective Study
Source: Front Oncol. 2021 Dec 15;11:795891. doi: 10.3389/fonc.2021.795891 (PMC8715708; doi:10.3389/fonc.2021.795891)
Supplement: Supplementary file 4 [file Table_3.docx]

Table SⅢ. Expression of ATG12, IGF2, and IRS2 in different groups of duodenal adenocarcinoma

gastric VS intestinal t test p value gastric VS pan t test p value pan VS intestinal t test p value

ATG12 1.9990 0.0101 * 0.0890 0.8494 1.9100 0.0121 *

IGF2 -2.1491 0.0291 * -2.2776 0.0291 * 0.1284 0.7852

IRS2 2.0473 0.0445 * 2.1570 0.0371 * -0.1097 0.8964

gastric VS intestinal, ratio of gastric type VS intestinal type; gastric VS pan , ratio of gastric type VS pancreatic type; pan VS intestinal , ratio of pancreatic type VS intestinal type *P < 0.05;
